# Comprehension of the Co-Operation of Professional Identity and Metacognition of English Teachers in Pedagogical Problem Solving

**DOI:** 10.3390/bs12020032

**Published:** 2022-01-31

**Authors:** Insuk Han

**Affiliations:** National Achievement Test Supporting Centre, Busan Metropolitan City Office of Education, Busan 47119, Korea; glueball@daum.net

**Keywords:** problem solving, professional identity, metacognition, professional development, narrative approach

## Abstract

Teaching is a metacognitive pedagogical problem-solving process. Teachers’ commitment to this process can be partly influenced by their professional identity (PI) in the pursuit of identity-congruent actions and identity verification. For these pursuits, teachers produce cognitive, affective, and behavioural responses, which are the representations of PI, and use metacognition by monitoring and regulating these responses for successful pedagogical problem solving. Teachers, in turn, improve their PI and metacognition. Based on a narrative approach, the problem-solving processes of five Korean teachers of English are explored along with their PI and metacognition operation. This exploration provides the rationales for the conceptualised co-operation mechanism of teacher PI and metacognition, and also reveals the possibility of its variation. Comprehension of the mechanism enables teacher educators and policy makers to establish specific plans and procedures for principled professional development or policy support. Thus, based on the conceptualisation and findings, systematic interventions via problem-solving-based teacher education and contextual support, which help teachers develop PI and metacognition, are discussed.

## 1. Introduction

Teaching or designing teaching is an iterative problem-solving process, including planning, implementing, and evaluating pedagogical strategies [1,2,3]. Working on these metacognitive activities [4,5,6], teachers monitor their cognitions, such as knowledge of person, task, and strategy, pedagogic actions, and related emotions, and they regulate these for the success of problem solving [7,8,9]. Thus, teaching is a metacognitive pedagogical problem-solving activity, and metacognitive teachers are likely to perform successful teaching [8,10].

However, not all teachers actively engage in solving pedagogical matters, and not all are successful at it. Their commitment to and direction of problem solving can be affected by their professional identity (PI) and its content/meanings activated in their particular contexts [11,12]. Given that people tend to perform identity-congruent actions [12,13,14], teachers can be motivated to solve their pedagogical problems, especially when the content/meanings of their PI highly regard exerting professional practice. Simultaneously, in order to achieve positive emotions [15], people desire their identity to be verified by others, by having others perceive them in line with their internal meanings of self [15,16,17]. Thus, when engaging in pedagogical problem solving, teachers aim to succeed in it to be recognised as professionals by others such as their students, colleagues, and administrators, so they are likely to (un)consciously activate their metacognition in the process of problem solving; identity activation includes the activation of related thinking procedures [14]. Using metacognition, they can manage their cognitions, emotions, and actions that constitute and represent their PI, and these processes increase the possibility of their successful problem solving [18,19].

Exercising metacognition while solving pedagogical problems, teachers can enhance their metacognitive skills [20,21] and develop their PI through reflections (which are metacognitive activities of monitoring) on and modifications (which are metacognitive activities of regulations) of the content/meanings constituting PI by learning from their findings [22]. Then, the developed PI and metacognition can result in changed practices, that is, improved pedagogical problem solving. In this way, teacher PI and metacognition can operate and develop together over pedagogical problem-solving processes.

Specific comprehension of the mechanism about the operations and relations of teacher PI and metacognition can help to partly explain why teachers address their pedagogical problems differently. It also reveals several rationales for developing educational interventions to support teachers to be effective problem solvers in actual teaching and learning situations. However, the conceptualisation of the operations of teacher PI and metacognition in combination and research to support the validity of such conceptualisation through the explorations of teachers’ teaching practices have not been fully addressed. Thus, in the present study, the researcher (a) conceptualises the mechanism by which teacher PI and metacognition co-operate over the pedagogical problem-solving process and (b) explores the pedagogical problem-solving processes of five Korean teachers of English in relation to this conceptualisation; the current study deals with teachers’ long-term problem-solving processes and their PI and metacognition operation which are embedded in them. On that basis, this paper (c) discusses several implications to assist teachers in developing their PI and metacognition through problem-solving-based professional development and related contextual supports.

## 2. Comprehension of the Operation of Professional Identity and Metacognition in Pedagogical Problem Solving

### 2.1. Professional Identity and Its Dynamics

Despite its different conceptualisations [23], the notion of PI seems to have evolved from a construct involving role-centred perception [24], as one of social identities [25], to including both social and personal sides of an individual [26,27] and context-related and transformable attributes [12,28], as defined and evolving through teacher interaction in relational contexts [11,29,30,31]. PI is thus regarded as multiple, (re)constructed, shifted, competing, and negotiated over personal, social, cultural, historical, and political contexts [32,33,34]. Comprehension of identity as close to a metacognition [35] and as an outcome of the engagement of multiple neural networks [36] adds to the conceptual complexity of PI.

With a teacher’s multiple affiliations and positions, teacher PI is constituted of several identities, such as person identity, national identity, (subject) teacher identity, and gender identity [17,33,34,37]. Depending on the contexts, particular identities actively operate [12] or are prioritised through identity competition [17,26,34,37]. The teacher tries to make negotiations among these identities and between these and contextual requirements, and the negotiated identities engage in directing related cognitions, emotions, and actions [26,34]. Thus, teachers’ cognitions, emotions, and actions are representations of their PI, which means that investigation of these is one of the practical methods that can be used to access the attributes and essence of their PI [33,34]; the current study focuses on analysing these components revealed in the teacher participants’ narratives in order to comprehend their PIs and related thinking procedures.

Identity contains relevant content [12,14] or a set of meanings that directs a person’s cognition, emotion, action, and sense of the world [26,34]. Identity competition is the interplay and negotiation of the content/meanings constituting it, and the development of identity is that of its content/meanings [32]. Identity content/meanings are a cognitive domain including what it personally and socially means to be a particular person [17]. These are about who I am, who I want to be, what to value, what to do, and what is expected of me, and these cover knowledge of person, task, strategy, world, procedures, norms, values, goals, and conditions in relation to the self [14]. Thus, when a teacher engages in problem definition, strategy design, strategy implementation, and its evaluation in a particular context, the salient identities and meanings constituting his/her PI can be activated, such as about what a good practice or an effective teacher means. As with belief systems, these can operate as lenses or standards through which teachers make sense of their world and make pedagogic decisions [38].

As the meanings constituting identity are (re)constructed by the person’s interaction with the context [12,14], teacher PI can transform through teachers’ experiences of pedagogical problem solving. Given that the transformed PI leads to the teacher’s changed practice [12,20,39,40], when professional learning focuses on reshaping teacher PI through problem-solving processes, it is likely to be successful.

### 2.2. Professional Identity as a Motivational Basis for Pedagogical Problem Solving

Identity activation in a given context accompanies the operation of related thinking procedures and actions [14,41]. It leads teachers to produce particular cognitive, affective, and behavioural responses towards their pedagogic environment [16,34]. In this sense, identity, or PI, seems to operate as a meta or meta-metacognitive power [34,35], which is a motivational basis for teachers’ pedagogical problem solving.

Interpreting their world through identity-relevant meanings based on metacognitive meaning comparisons (monitoring), people pursue identity-congruent actions, or what they want to do [42,43], as they believe this is right and true [12,14]. Therefore, if teacher PI includes the meanings that value professional practice and they recognise the possibility of identity-congruent actions in their context, teachers can engage in solving pedagogical problems and be likely to succeed in solving them. Then, they feel meaningful with themselves and their act, even when the problem is difficult to deal with [12,13,14]. In this way, teachers realise their autonomy, by which they can enjoy what they do [43]. However, when they recognise the difficulty of identity-congruent actions, they may disengage themselves from problem solving [36] or rather perceive the need for striving [14] or attempt meaning negotiations by regulating their cognitions, emotions, and actions [7,17,43]. These different metacognitive processes may bring them different outcomes.

People’s desire for identity verification also influences their decisions and actions. People want others to perceive them in line with their internal meanings of self, by which they can gain positive emotions and self-esteem [15,17] and prove their competence in their relational context [42,43]. Thus, when teacher PI contains the meanings that orient their performance of professional practice, teachers may endeavour for the success of problem solving in order to be recognised as effective teachers by the students, administrators, and colleagues. However, if the internal and external meanings of effective practice are not matched, teachers may try to reconcile them by regulating their practices and/or internal meanings [14,15,17,34] and even related emotions [44]; when the gap is significant, they may hesitate to strive for reconciliation [34]. Such an (metacognitive) endeavour and the following regulated/negotiated aims, pedagogies, and practices increase the possibility of successful problem solving [18,23], that is, identity verification.

In sum, effective teachers with a developed PI seem to mean that (a) their PI involves meanings that value professional practice; (b) they are motivated to solve pedagogical problems; (c) they can context-sensitively activate the identities and meanings, based on multiple meaning comparisons and negotiations; and (d) using metacognition, they monitor and regulate their cognitions, emotions, actions, and contextual factors for successful problem solving.

Based on these comprehensions of teacher PI, the next section addresses how teacher PI and metacognition interact and develop together over pedagogical problem-solving processes.

### 2.3. Operation of Metacognition and Professional Identity in Pedagogical Problem Solving

Metacognition, which is cognition about cognition [45], comprises monitoring and control of cognitive processes for learning or problem solving [46]. Monitoring is assessing the progress of a cognitive activity, and control is regulating the cognitive activity. These activities include competencies in attention, inhibitory control, conflict resolution, emotional regulation, time management, error correction, strategy selection and assessment, and enquiry of various meanings for the evaluation of processes and outcomes, by the relation of one’s knowledge to other information [44,47,48]. Thus, metacognition is in sum the critical awareness and regulation of cognitions, emotions, and actions, based on the multiple meaning comparisons and negotiations in the process of task performance.

Given that teaching is an iterative problem-solving process [1,2] that is essentially metacognitive [5], teachers use metacognition daily through multiple pedagogical meaning comparisons and negotiations, that is, monitoring and regulations. Their metacognition has a significant influence on each stage of problem solving, especially operating with their PI, as follows.

When defining the pedagogical problems, teachers monitor their current classroom situations comparing their perceptions with their meanings about desirable classrooms [49,50] and focusing on important, identity-relevant information [41,51]. Through this process, they can (un)consciously judge the availability of their identity-congruent actions [14]. When they perceive the gaps, they identify the problems and make representations of their problems through semantic or visual coding [52], considering the initial state, goal state, operators, and constraints [53].

By relating the represented information to relevant internal meanings, teachers design their strategy. They can retrieve or select their meanings about domain-specific knowledge or epistemic beliefs [22]; use heuristics for convenience [53]; apply analogies referring to their past experiences [49,52]; and attempt to formulate a new strategy by associating their knowledge [54].

Then, teachers can practise the strategy. As classroom realities frequently produce unpredictable situations [7], the initial implementations are liable to be pilot studies. While implementing, teachers evaluate the effect of their strategy by comparing their findings with their represented goal state [49]. When discovering matches, teachers can confirm their strategy and related meanings and consider the transferability of these [22]. Otherwise, they may experience frustration, and enquire the validity of their goals, strategies, and applied meanings, or taken-for-granted beliefs [55]. This is an identity crisis. They may try to overcome the cognitive and emotional crisis by metacognitive control [56,57].

Repeating experimentations and modifying their strategy, teachers become more skilled in metacognition including self-reflection [20,21] and establish new schemas with increased reliability of their findings [52], which may constitute part of the pedagogic meanings constituting their PI. With this transformed PI, teachers can, in turn, perform improved pedagogical problem solving [20,58].

**Figure 1:**
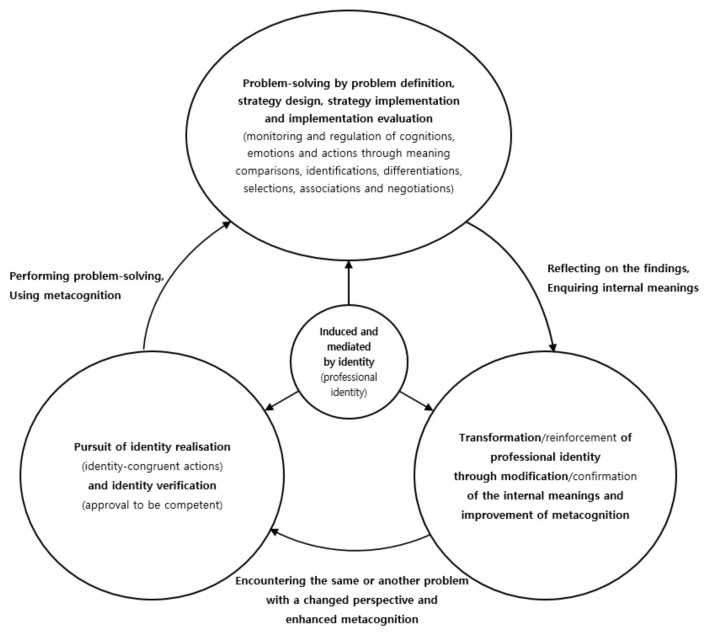
Co-operation and co-development of teacher professional identity and metacognition over the pedagogical problem-solving process.

Thus, it seems that teacher PI and metacognition operate and develop together organically through pedagogical problem-solving processes. See Figure 1 for a summary of this mechanism.

As the figure indicates, as teacher PI and metacognition are in a virtuous cycle, in an endless loop of daily pedagogical problem solving, the qualities of PI and metacognition can be proportional. Thus, teachers who have developed PI are likely to be those who are motivated to use and competent in using metacognition. However, not all teachers experience this developmental cycle. The next section considers in what ways teachers with different qualities of PI and metacognition perform their pedagogical problem solving differently.

### 2.4. Pedagogical Problem Solving of Teachers with Different Qualities of Professional Identity and Metacognition

Teachers with developed PI and metacognition have an advantage in terms of comprehending problems, with their competency in comparisons of what they perceive with what they know [5] and selective attention [41]; in goal setting, with their ability in comparisons and negotiations of meanings; and in strategy design, with their sufficient domain-specific knowledge/meanings [59,60]. If their strategies are ineffective, they may create new pedagogies by context-sensitively activating their identities and meanings and performing further negotiations between these and external meanings.

Such teachers may overcome their failure by controlling their emotion and make practical efforts for resolution [56], transforming the crisis into a chance for professional development [57]. Thus, operating metacognition of inductive reasoning [61], they may analyse, enquire, and correct their problem definitions [49] and consider alternatives [62]. In these senses, it seems that they have a strong tendency to elaborate themselves and to integrate new experiences [43], and their PI includes learner and researcher identities [55] for effective problem solving.

Meanwhile, teachers with less developed PI and metacognition may be inactive in pedagogical problem solving and reject meaning negotiations. As they have less structured schemas [2,60] and are unskilled in using metacognition, they may notice only the surface features of the problems [2] and have difficulty in defining problems [60,61] and designing solutions [2]. Clumsy in context-sensitively activating their identities and meanings and in understanding situations, they may not produce appropriate goals and strategies. As they cannot establish specific assessment criteria, they may depend on their feelings [57]. When failing in problem solving, they may not improve the situation because they are unskilled in interpreting the causes [2], that is, in metacognitive reflection. They are novice problem solvers.

Nonetheless, if their PI comprises learner or researcher identity, they may possibly develop into those with developed PI and metacognition through repeated pedagogical experimentations [63]. In fact, novices are reported to carefully scrutinise their situations, try out innovative methods [13,60], and try to develop professionalism in the professional community [64]. With this activeness and flexibility, they have the potential to be better problem solvers.

Based on the current comprehension of teacher PI and metacognition, the processes of pedagogical problem solving can be complicated, as in Appendix A. Considering teachers’ real-time identity shift and metacognitive monitoring and regulation of their cognitions, emotions, and actions [7], the illustration can be more multi-layered. However, given that teacher motivation, behaviour, and development can be significantly affected by the pedagogical context [12,43], there seem to be no teachers with consistent advantages in actual problem-solving situations [5]; see findings and discussions.

The next section introduces the research design of the current study. Then, the problem-solving processes of five Korean teachers of English in high schools are presented in relation to the operation of their PI and metacognition. This exploration will provide both the rationales and variability of the operation mechanism of teacher PI and metacognition described in Appendix A.

## 3. Research Design

### 3.1. Participants and Settings

Every teacher has various identities and a particular level of metacognition. Nonetheless, as contextual varieties significantly influence identity activations [12], the researcher limited the target to teachers who were teaching or who had taught in academic high schools to draw out more comparable and thematic data. While academic high school teachers were commonly required to adopt the national English curriculum framed within the communicative language teaching (CLT) approach, the Korean Scholastic Aptitude Test (KSAT) was formatted to mainly measure the listening (35%) and reading (65%) skills of the students. Thus, according to the participants as well as relevant studies [33,34], most (English) teachers in academic high schools could not disregard their students’ test preparation and the use of the grammar translation method (GTM) for reading classes. This means that the teachers who were teaching or who had taught in academic high school were likely to experience or had experienced multiple meaning conflicts and negotiations or crises of their identity or the need to transform or adapt their roles, pedagogic endeavours, and identities through related metacognitive thinking procedures.

Thus, 32 high school English teachers who participated in one day of a test skill development programme in Busan Metropolitan City, South Korea, were informed of the research. Based on their agreement and experience of teaching in academic high schools, 12 of them were sent an email by the research outlining the research aims and procedures. Four of these teachers agreed to participate, and one of them, Eun-ju, introduced her colleague, Sumi, to the researcher with her consent. Ultimately, five teachers participated in the research (see Table 1); pseudonyms were formed by the participants and they were used throughout the study for anonymity and confidentiality. The limited number of the participants is a limitation of the current study, but, by using a qualitative approach, it enabled the researcher to deeply comprehend the mechanism of the operation of teachers’ PIs and metacognition in relation to the relevant conceptualisation. While a large number of participants may result in quantitative studies that reveal several content/meanings that constitute PI [65], the qualitative approach that targets a relatively small number of participants can often lead to the effective exploration of the content/meanings and the change of teachers’ PIs or relevant thinking procedures [66].

The four female participants were teaching in state high schools; Sumi and Eun-ju were teaching the same grade of students in the same school. One male was teaching in a private high school. The teachers in state schools were required to rotate their workplace every three to five years until the retirement age of 65, while those in private schools were required to work in the same school, but with less job stability. This system can be an influence on teachers’ identity activation and development as well as the extent of their use of metacognition, and thus their teaching practices. 

According to their years of teaching, teachers may show different forms and activation of PI and metacognition. Nonetheless, given that the current study tries to reveal and address the outlines or shared patterns of teachers’ pedagogical problem-solving processes and the possibility of their variation, and the operation and development of PI and metacognition embedded in the processes, the study does not sift the participants by the years of teaching experience; refer to [63] for the comprehension of the operation of PI and metacognition of experienced English teachers, and see [67] for the comparison of the operation and development of PI and metacognition between an experienced and a novice teacher.

### 3.2. Methodology and Data Collection

Teacher narratives have been used in various studies of teacher identity with its appropriateness of disclosing the attributes and development processes of teacher identity through teachers’ interplay with their personal, social, cultural, historical, and political contexts [68,69,70]. As their stories include their knowledge, perspectives, experiences, tensions, resolution, changes, and integration of theory and practice and of the personal and professional worlds [70] and their emotions [71], teacher narratives represent their PI and daily pedagogical problem solving in an intermingled way. Furthermore, verbalising their stories, teachers can restructure their scattered memories through reflective dialogue with themselves and others, and by stepping back from themselves and their experiences [72]. Thus, narrating is a process of exploring the self and meaning making about who they are [73]. Meaning construction through the narratives is teachers’ metacognitive activities of organising their experiences to be reportable, through which the taken-for-granted meanings constituting their PI and implicitly performed metacognitive meaning comparison and negotiation processes in their pedagogical problem solving come to the surface. Therefore, teacher narratives are the right passages to investigate both the identities and metacognition of teachers. Thus, the following methods were applied.

Three teachers (Yuna, Hei-jin, and Hee-jun) participated in interviews twice for one to two hours each, with an interval of two to six months. The interview schedule was determined by their availabilities. In the first interview, they introduced their demographics and school environment. Then, they narrated their teaching experiences according to the researcher’s semi-structured questions, as the less structured questions can let them recollect their memories with less bias [74]. The questions sought their recent and recent-past teaching procedures, (un)satisfying teaching experiences, endeavour for pedagogic improvement, and ideas of desirable pedagogies and their changes; see Appendix B. The questions were previously applied in researcher’s other studies [63,67] of the exploration of English teachers’ PI and/or metacognition so they were sufficiently validated. Since questions that require too much reflection or a description of the process of overcoming challenges can cause the narrators to restructure their stories to include a happy ending [73], the researcher tried to let them describe their experiences as they were by not inquiring about any conclusions. Questions seeking clarifications of the rationales of their cognitions, emotions, and actions were frequently applied in order to disclose their PI and related metacognitive thinking procedures. This also helped reveal different cognitions and emotions implied in the teacher participants’ similar behaviours [60]. In the second interview, the researcher asked the participants to clarify their previous statements and to review and confirm the interpreted data through member checking [75,76].

Su-mi and Eun-ju preferred to be interviewed together. Though their participation in individual interviews might have been the best scenario, the researcher prioritised the participants’ suggestion as an ethical matter. They were not in any hierarchical position in their workplace and their narratives were critical and straightforward in describing their cognitions, emotions, and actions in the process of pedagogical problem solving. They also provided the researcher with some written data—Su-mi’s memos and Eun-ju’s diary, including their daily in-context pedagogical problem-solving processes over six months. As they were teaching the same grade of students in the same school, their practices were interrelated. Thus, their data are presented together in the current study. Their data also revealed the potential of teachers’ daily, flexible, and spontaneous interactions. The implications of their interaction for teachers’ professional development are covered separately in another paper [67].

All the data were produced in Korean for the participants’ convenience. The interviews were voice recorded based on their agreement, which were transcribed into Korean and translated into English by the researcher for data analysis and presentation.

### 3.3. Data Analysis

The data analysis process was a series of metacognitive activities for the researcher. The analysis began when the researcher gathered the data and the second analysis during the transcription, translation, skimming, and scanning of the records. In these processes, comparing what the participants reported with the research aims and questions, the researcher constantly monitored if the interview questions led the participants to specifically reveal their stories of pedagogical problem solving and the operation of their PI and related metacognitive thinking procedures. In these initial readings and the following reviews, the researcher focused on analysing the individual as well as the shared cognitions, emotions, and actions of the teacher participants, given that these are the representations of PI, and that the essence of PI can be accessed through an exploration of these components [33,34]. The teachers’ (un)conscious recognitions and manipulations of these components are metacognitive activities to be marked out in the current study.

In the third attempt, the researcher reorganised the data in chronological order, as semi-structured questions did not lead the participants to narrate their experiences in a linear way [69]. Then, the researcher could inductively reformat their problem-solving process into four procedures, considering the attributes of in-context teaching situations, the observability and reportability of PI and metacognition operation, and the changes of the engaged cognitive and metacognitive activities: problem definition (identifying problems and establishing goals), strategy design (developing strategy and materials), strategy implementation (applying and reshaping the strategy), and implementation evaluation (forming new meanings from the findings). According to these four categories, the practices including relevant cognition, emotion, and actions of each participant were summarised in an Excel sheet.

In the fourth endeavour, the activations of the identities/meanings and metacognition were more specifically disclosed by a thematic approach [77]. The teacher participants’ recognition and disclosure of their roles, positions, pedagogic values, and relevant cognitions, emotions, and actions were captured as representations of their PI, that is, the identities and their meanings constituting PI [33,34,78,79]. The self-awareness, reflections, and transformations of these components representing their PI were regarded as representations of their metacognition. Using conceptual grouping based on the inductive reasoning on the summarised data, 12 subcategories emerged (see Table 2). Each subcategory involves both the teacher participants’ PI activation and metacognitive monitoring and control, though the quality of these may be different from one another. Based on these subcategories, the researcher further detailed the participants’ metacognitive activities. People cannot easily explicate their own metacognition in their experiences, because metacognition often operates implicitly as well as explicitly [80] or fused with cognition over problem-solving processes.

In the fifth analysis, the researcher focused on drawing out themes to discuss by summarising and arranging all the participants’ experiences into a matrix (see Appendix C). By ranging the participants vertically and the four procedures of problem solving horizontally, the researcher sorted out the eight individual themes by participants and their seven common themes by problem-solving procedures. The sixth analysis was performed during presentation. While presenting the data and its interpretations in relation to the relevant literature, the researcher re-examined if the categories were appropriately educed and applied, and the themes fully described the activations of PI and its meanings, the interplays between different meanings and the role of metacognition in these processes for problem solving. Finally, the member checking [74,75] confirmed the value and precision of these interpretations.

## 4. Co-Operation of Professional Identity and Metacognition of Korean Secondary School Teachers of English over Their Pedagogical Problem-Solving Processes

Authors should discuss the results and how they can be interpreted from the perspective of previous studies and of the working hypotheses. The findings and their implications should be discussed in the broadest context possible. Future research directions may also be highlighted.

### 4.1. Su-mi’s and Eun-ju’s Experiences

#### 4.1.1. Problem Definition

Su-mi and Eun-ju identified problems by perceiving their students’ low concentration and academic levels. Firstly, they tried to understand the cause of their problems, reflecting on their current lessons. With her varied domain-specific knowledge as an experienced teacher [59,60], Eun-ju’s reflection was more precise and analytical [2] than Su-mi’s. She recognised her bad habit of not giving students sufficient time to prepare for their answers and her use of uneasy English expressions.

Making negotiations between their internal meanings (of increased learner attention and comprehension) and external meanings (of learner needs for KSAT preparation and curriculum requirement about exercising CLT), they aimed to enhance students’ reading competencies using the CLT approach and to increase learner participation; they individually specified different sub-goals.

#### 4.1.2. Strategy Design

Su-mi and Eun-ju incorporated their knowledge of self, learners, problems, strategies, and curriculum into strategy design. Based on her recent successful pedagogic experience, Su-mi planned to teach new words before text reading. Eun-ju planned to teach them while reading according to her old repertoire [2,8]. Based on the meaning negotiations, they decided to partly apply GTM; their simultaneous pursuit of GTM and learner participation reveals the inconsistent meanings in their PI.

*Eun-ju: Teachers can be given more discretion in … developing learning content and activities by escaping from GTM. … [However,] we, including our students, tend to think that we did nothing without translating the text. … Isn’t it OK to combine several advantages of different pedagogies [including GTM] if it makes learning take place?*

Through communications, they planned to include group work, video materials, and worksheets. Eun-ju specified these. Su-mi made the video materials, learning to edit them with her learner identity activated. Eun-ju created the worksheets, during which she enquired her teaching methods through metacognitive monitoring. They shared their materials but modified them according to their own preferences.

#### 4.1.3. Strategy Implementation

Su-mi applied the strategy, but she was frustrated with the results. The video material was too long. Learner groups organised by achievement levels made the students feel uncomfortable. She needed more skills and experiences in classroom management [2] and in regulating her cognition and emotion.

*Su-mi: I would turn back to my [teacher-led] mode. For group work, teachers should be able to control their students, but I can’t. … They made a shambles of the class and I’m not good with words.*

Based on her inductive reasoning [61] and Su-mi’s teaching, Eun-ju let her students construct their own groups, helped individual learning, and shortened the learning content. She then found increased learner participation and concentration. Eun-ju’s acceptance of new findings from strategy implementations and effort to contextualise her strategy disclose the activation of her metacognition and learner and researcher identities.

#### 4.1.4. Implementation Evaluation

Su-mi and Eun-ju repeatedly implemented and reshaped their strategy. Comparing the findings with their goals, both concluded that their methods were generally effective, but needed more communicative aspects. Su-mi formed a new meaning that high-achieving female students do not enjoy group work and she wanted to develop new methods. These analytical responses reveal her developing PI and metacognition through problem solving and increased confidence from her partial success [43,81]. Reflecting on the causes of her partial failure [2], Eun-ju judged that GTM limited communicative lessons, so she considered an alternative [62] using analogical transfer [52] and creativity [82].

*Eun-ju: I thought about how I constructed classroom activities when teaching in a middle school. I was flexible and creative and gained ideas from my surroundings. … Yesterday, watching a TV show in which a reporter and a movie star were having an interview, I came up with an activity by which students adapt their text into a script for role-play.*

It seems that Eun-ju had a relatively developed PI and metacognition compared to Su-mi, but both reshaped these through learning from their problem-solving processes.

### 4.2. Yu-na’ Experiences

#### 4.2.1. Problem Definition

Yu-na valued learner participation and teaching language skills and cultures; these were reconcilable with the national curriculum principles. Comparing these meanings with the students’ expectations of the KSAT preparation in an academic high school, Yu-na problematised her situation.

*When teaching high school seniors, … I deliver knowledge to them like a robot. … I feel that they pursue efficiency by the pressure of test preparation and select out the extract from me for the test. I’m very unsatisfied with this unavoidable lecture-based class.*

Recognising the non-negotiability of meanings and prioritising her identity-congruent actions [12,13,17], she moved to a vocational high school, where the students in general did not prepare for the KSAT.

#### 4.2.2. Strategy Design

In the vocational school, by incorporating compatible meanings, such as learner needs for learning communicative skills, school curriculum about teaching practical English and her existing pedagogic meanings, Yu-na designed the lessons, in which the students organise travel plans. This strategy was inspired by her reading of world cultures, that is, association of relevant information [2].

#### 4.2.3. Strategy Implementation

By implementing her strategy with different classes, Yu-na observed that students actively explored necessary information, prepared a presentation themselves, and practised English writing and speaking, all while enjoying their learning. Comparing these responses with her aims, she judged that her strategy was effective in general.

*A group selected Japan. Those incompetent in speaking English prepared a kimono and tried to use simple English. … They seemed to feel the pleasure of participation, while having used Konglish. … I was amazed by their hidden talents.*

Yu-na learned that ‘when teachers set the right stages, students perform well’ and she confirmed her meanings of learner-centred, theme-based learning.

#### 4.2.4. Implementation Evaluation

Through repeated experimentations, Yu-na formed a schema [52], or a pedagogic meaning, that English classes should ultimately help students’ self-realisation. She thought this meaning would not be realised in academic high schools with the contextual constraints. In this way, Yu-na reshaped her PI, monitoring and regulating the value of the meanings that constitute it.

### 4.3. Hei-jin’s Experience

#### 4.3.1. (Episode 1.) Problem Definition

Hei-jin valued learner participation, autonomous learning and practical English learning. Teaching in a suburban academic high school, she perceived the administrators’ needs for test preparation, but students’ low interest in learning. Based on this understanding, she aimed to improve learner interest and autonomy by providing them with various classroom activities while using the text materials for test preparation.

#### 4.3.2. (Episode 1.) Strategy Design

Trying to reconcile the requirements of the school, learners, and curriculum and her pedagogic meanings through negotiations, Hei-jin designed several activities, such as writing phrases to fill in the blanks, making a follow-up story, and exercising pronunciations by team competition. She formed these methods by analogy [49] and articulation of old representations with novel representations [82], or her creativity. This approach evidences her rich pedagogic meanings and activation of metacognition and PI.

#### 4.3.3. (Episode 1.) Strategy Implementation and Implementation Evaluation

Implementing her strategy and observing learner participation, Hei-jin judged that her strategy was effective and confirmed her meanings about learner-centredness [83].

*While doing the tasks, the students learn something interactively. … They listen to their friends, though they don’t listen to me. … It’s rather bothersome to prepare for such classes but … I felt rewarded after … observing that they do something.*

Hereafter, Hei-jin taught in an academic high school in a new town, where she context-sensitively activated the identities and meanings that constitute her PI and negotiated external meanings from the non-coherent curriculum.

#### 4.3.4. (Episode 2.) Problem Definition

In her new context, the students required test preparation and were familiar with lecture-centredness, though not favouring it. Associating this comprehension with her pedagogic meanings, Hei-jin aimed to control learner passiveness and distraction.

#### 4.3.5. (Episode 2.) Strategy Design

By accepting her students’ assumption of ‘reading by translation as learning’ and negotiating the expectations of her colleagues, Hei-jin planned to conduct a GTM-based lecture (for 30 min) and to have the students autonomously work on worksheets that contain translation activities and word quizzes (for 20 min).

*Others say, ‘Is your method realistic in the current context, where the KSAT is focused on reading?’ … I teach [my students] the same content but in ways different from other teachers’. … Students rather feel and learn something in the process of self-reading.*

#### 4.3.6. (Episode 2.) Strategy Implementation

From repeated implementations, finding that the students could not easily solve the given questions, Hei-jin reshaped them and increased individual feedback. With their poor management of the worksheets, she checked the management by a checklist; learning from the findings and creating new methods reveal her developed metacognition and learner and researcher identities. She emphasised teachers’ patience and flexible strategy modifications, that is, monitoring and regulations of cognition, emotion, and action [12,44].

#### 4.3.7. (Episode 2.) Implementation Evaluation

Identifying her students’ enhanced concentration and autonomy, Hei-jin confirmed her strategy and formed a meaning [52] that regular inspections of the students’ learning can lead to their self-directed learning. This implies that her PI developed from problem-solving experiences [11,22]. Her constant monitoring and regulations seem to have also resulted in metacognitive students [7].

### 4.4. Hee-jun’s Experience

#### 4.4.1. Problem Definition

Teaching in a private academic high school, Hee-jun valued knowledge delivery; this meaning was reconciled with the goal of his institute. However, he stated that without the needs of the students and administrators for test-preparation, lessons should involve learner participation, by which students learn themselves and form a worldview to be cosmopolitan. This implies that his PI may contain inconsistent meanings.

*Without a performance-oriented goal … I can derive some ideas from the students. … When reading, letting them think about the writer’s voice, letting them have a discussion, and having them write a new text based on this. … Then they’ll spontaneously recognise reading skills without my instructions. … I hope they feel some pleasure [from learning].*

By integrating the conflicting internal and external meanings [5], Hee-jun aimed to develop test-preparation lessons, including simple activities to enhance learner participation.

#### 4.4.2. Strategy Design

Based on his experiences, Hee-jun had an accustomed pedagogic repertoire [7,60,84]: (a) letting students recognise the aim of learning particular knowledge, (b) explaining the knowledge structure to them, and (c) letting them apply their learning through worksheets or related activities.

#### 4.4.3. Strategy Implementation

Based on the eye contact with his students and feeling that they followed the lesson well, Hee-jun judged that his strategy was generally successful. When perceiving some signals of misunderstandings, he attributed these to his misinterpretations of the learner levels. Then, he partially modified the content and material, but he did not substantially change his practice. This implies that while he monitored autonomously, he was unskilled in regulating problem definition [49] and strategy design [7] and/or his contexts had him prioritise his employee identity [2,12] impeding his experimentations.

#### 4.4.4. Implementation Evaluation

In order to address his discomfort caused by the limited identity-congruent actions, Hee-jun practised task-based learning (TBL) right after his students’ exams. Nonetheless, with his inconsistent internal meanings and the irreconcilability between his new method and the learner needs, he felt uncomfortable with his alternative; he monitored, but could not regulate his meanings.

*Feeling ashamed of my current practice, … after the mid- or final exam, I try to break the convention and console myself. I throw away two to three hours doing ‘extravagancies, [that is, TBL]’. … These expressions are very contradictory. Though thinking something ideal, I call it vagarious. … I’m afraid that my students would say of me, ‘He wastes time.’*

Hee-jun could not verify his identity as a professional [15]. He turned back to his accustomed method, as this guaranteed his partial identity verification and positive feelings through the partial reconciliation among his cognition, emotion, and action. By adopting his external meanings and activating his meanings reconcilable with them, his development of PI and metacognition through meaning negotiations was limited.

## 5. Implications for the Development of Professional Identity and Metacognition of Language Teachers

From the exploration of the teaching processes of the teacher participants, it is found that language teachers’ PI and metacognition co-operate through their pedagogical problem-solving processes, and they are reshaped through the teachers’ monitoring and regulations of their cognitions, emotions, and actions and even sometimes monitoring and regulations of relevant external meanings such as the requirements of the students, administrators, and curriculum. These findings become in general the rationales of the conceptualisation of the co-operation mechanism of teacher PI and metacognition addressed in the previous sections. Meanwhile, identification of the significant power of the contextual constraints that limited pedagogical meaning negotiations and experimentations of Yu-na, Hei-jin, and Hee-jun implies that the mechanism can operate rather variably, closely or distantly orbiting around the lines depicted in Appendix A. From this understanding, implications for teacher education can be discussed.

From the exploration of the co-operation mechanism of teachers’ PI and metacognition through pedagogical problem-solving processes, the ideal state for teachers’ well-processed practices seems to occur (a) when teachers’ PI includes learner and researcher identities and the meanings that value professional practice (then, for identity realisation and verification, teachers try to solve their problems using metacognition) and (b) when teachers’ internal and external meanings are negotiable, and so their negotiated goals and strategies satisfy both others and themselves. However, as revealed, given that teachers have different qualities of PI and metacognitive skills and face different contextual constraints, systematic interventions seem necessary to help them realise negotiated pedagogies and develop their PI and metacognition through problem-solving experiences. Thus, in the next section, the researcher discusses ways to lead teachers to experience multiple meaning negotiations and development of their PI and metacognition through programmes based on problem solving and contextual supports.

### 5.1. Problem-Solving-Based Teacher Education

#### 5.1.1. At the Stage of Problem Definition

Su-mi was incompetent in problem definition and strategy design with her insufficient domain-specific knowledge and metacognitive skills, while the others were relatively skilled in these with their recognition of their own pedagogic meanings and contextual requirements [2,60]. Hei-jin solved her problems based on her comprehensive understandings of her meanings and situations. Yu-na and Hee-jun, while they could not improve their situations, understood their situations and problems through comprehensive monitoring. In this sense, possession of various pedagogic meanings and competencies in integrating different meanings seem to be partial conditions of a developed PI and metacognition.

Thus, firstly, teacher educators need to let teachers recognise the importance of metacognitive monitoring and regulation through discussion [18] and of performing professional practice for quality education, Then, they can lead teachers to acquire knowledge of pedagogy, subject and curriculum [85] and to associate these with their internal and external meanings to define various problems in different contexts through problem identification, cause interpretation and goal setting. Issues can be selected based on the survey about the teachers’ internal meanings about good practice [13]. In the beginning, teachers can address analogous or isomorphic problems to increase self-regulation [12] and progress to more complicated ones. This will help novices like Su-mi feel a sense of accomplishment. Then, teachers can learn to clarify their goals with the educators’ questioning (see [60]) and specify subgoals for their detailed evaluations of strategy implementation later [18]. When having difficulty in these activities, teachers can share their ideas for the reference of different meanings and knowledge, as did Su-mi and Eun-ju in their developmental interaction.

#### 5.1.2. At the Stage of Strategy Design

Eun-ju and Hei-jin created negotiated pedagogies by incorporating their meanings of communicative lessons and external meanings for test preparation. Through these pedagogies, they pursued both identity-congruent actions [12] and identity verification [17]. Whether to more pursue the former or the latter was regulated by their activated identities and meanings and the negotiations between these and the contextual requirements. In a suburban school, Hei-jin realised more of her meanings and in an urban school, more of test preparation. Meanwhile, prioritising her meanings of the CLT, Yu-na moved where contextual requirements were modest. Activating his employee identity, Hee-jun generally maintained the practices externally required. Thus, for more professional practice, that is, design and implementation of effective strategy, teachers need to learn to perform balanced meaning negotiations.

In order to produce negotiated pedagogies that broadly satisfy both teachers and other stakeholders, teachers can be trained to explore the expectations of different stakeholders through research and inductive reasoning. Then, they can exercise comparing their internal and external meanings and prioritising or deferring certain identities or meanings in various situations. Negotiating different meanings, teachers can develop contextualised pedagogies as well as their metacognition and creativity [18]. As pedagogical problems have no fixed solutions [5], having teachers share their representations can be effective for them to consider various strategies [18] and raise greater metacognition [60]. Beginning teachers can learn about various representations from their seniors, as did Su-mi. Stimulated by the beginners, experienced teachers may enquire their meanings, as did Eun-ju.

#### 5.1.3. At the Stage of Strategy Implementation

Facing unexpected outcomes from strategy implementations, Su-mi abandoned incontrollable pedagogic methods with her clumsy metacognitive regulations of her cognition, emotion, and action (as well as those of her students); she gradually overcame her frustration repeating experimentations and communications. Others compared what they were doing and what they intended to do. Learning from this monitoring, they contextualised or confirmed their strategies and meanings. Eun-ju and Hei-jin improved their partial failure by reflecting on their strategies [49], interpreting the causes [2], and modifying their strategies [62]. In this way, they overcame frustrations and developed professionalism, which reveals their developed and developing PI and metacognition.

From these findings, the ways to help teachers improve their performance and overcome failure seem to let them exercise in refining their strategy through monitoring and regulations of their cognitions, emotions, and actions and analysing learner responses. For these activities, teachers can make their own checklist based on their (sub)goals, learn to collect different findings [51], and compare these through reflections [58]. On their failure, teacher educators can let them re-examine their problem definition and strategy design, providing several questions (see [18]) and having them explicate the causes of failure [2]. Collective deliberations on the video-recorded lessons [11,60] can also help reflections. While interacting, teachers can consider different findings and alternatives and regulate their cognition and emotion by externalisation.

#### 5.1.4. At the Stage of Implementation Evaluation

From the repeated implementations, reflections, and modifications of strategies, the teachers gradually developed their metacognition [20,21] and incorporated their findings into their pedagogic meanings through which their PI evolved [22]. Su-mi formed new meanings with situated knowledge and revealed more precise reflections on her practice with increased confidence from partial success [83], while Eun-ju focused more on what to improve [49], questioning her repertoires [60]. Yu-na, Hei-jin, and Hee-jun expanded their pedagogic meanings, from the focus on knowledge delivery to self-directed learning. On these processes of PI and metacognition development of the participant teachers, their learner and researcher identities seem to have played critical roles: (a) leading the continuations of problem solving and (b) contributing to the activation of metacognition for meaning modifications, that is, PI transformation, through pedagogical experimentations.

Therefore, in order for them to learn from their problem solving and constantly develop their PI and metacognition, teachers need to be educated to perform reflections on both their own meanings and relevant external meanings and accomplish meaning modifications working as a researcher and a learner. This will be available by having them consider the transferability of their findings in different contexts through various experimentations [86]. Leveraging different identities in the shifted contexts [87], they can activate the thoughts, actions, and emotions expected of teachers, learners or researchers and experience different modes of metacognition. When repeating successes, teachers can form reliable pedagogic meanings to improve their PI. When repeating failures, they need to learn about the implications of the issues for their identity development [13] or self-development [43] and be encouraged to perform meta-metacognitive monitoring and regulations [62]. These activities should be planned as a long project [88], particularly for the meaning changes of experienced teachers, considering their adherence to old repertoires [7].

### 5.2. Contextual Supports for the Development of Teacher Professional Identity and Metacognition

Yu-na’s, Hei-jin’s, and Hee-jun’s difficulties in meaning negotiations in high schools, where the students and administrators pursued test preparation, limited their pedagogic improvement through experimentations. With the contextual constraint [12], Yu-na attempted to escape her problematic situation; Hei-jin relinquished part of her meanings; and Hee-jun practised externally preferred practices for partial identity verification while feeling antinomy maintaining incompatible meanings. Thus, to facilitate teachers’ motivation for and availability of problem solving, meaning negotiation, further experimentation, and the subsequent development of their PI and metacognition, contextual support seems necessary.

One of the main causes of the rigid meaning negotiations was found in the non-coherent national English curriculum, by which teachers are guided to practise the CLT approach, and students are supposed to take the reading-focused KSAT; similar situations are observed in China [89], Japan [37], and Vietnam [90]. As students’ KSAT score influences their university entrance, which then affects their economic status and marriage, the expectations of the students, parents, and administrators for test preparation are strong in Korea [91,92], and teachers cannot disregard them. Thus, if the form of the KSAT changes into measuring balanced language skills so being in line with the curriculum principles, the stakeholders may focus on learning the practical aspects of the English language. Then, English teachers will find the increased negotiability of meanings and possibility of experiment with various pedagogies.

School administrators can also support teachers to develop their PI and metacognition. By guaranteeing teachers’ autonomy [7] and creating a school culture where teachers can perform individual or collective pedagogical experimentations [86], they can help teachers monitor and regulate their own cognitions, emotions, and actions and solve their problems through communications and reflections.

## 6. Conclusions

This study conceptualised the co-operation mechanism of teacher PI and metacognition over teachers’ pedagogical problem-solving processes and explored the problem-solving processes of five English teachers in order to give the rationales of the conceptualisation. In sum, for the pursuit of identity-congruent actions and identity verification, teacher PI, especially when it contains the meanings that value professional practice, can motivate teachers to solve their pedagogical problems. In the process of metacognitive problem solving including problem definition, strategy design, strategy implementation, and implementation evaluation, teachers use metacognition, monitoring and regulating their cognitions, emotions, and actions. This is because using metacognition increases the possibility of successful problem solving, which, in turn, enables the realisation of teachers’ identity relevant actions and identity verification. In this experience, teachers exercise and enhance their metacognition and reshape their PI by learning from the findings and modifying the meanings constituting PI. This can lead to their improved pedagogical problem solving. However, depending on the quality of teacher PI and metacognition, their prioritisation of particular identities/meanings in their contexts, and the negotiations between these identities/meanings and their external requirements, teachers reveal different problem-solving processes alongside different cognitive, emotional, and behavioural responses. 

Comprehension of this mechanism explains why teachers behave differently in the face of pedagogical matters, and reveals the cognitive and metacognitive components to be considered in the process of designing teacher education programmes. The programmes which aim to reshape teacher PI and metacognition will result in teachers with changed (meta)cognition, emotion, and action, namely improved professionalism. This will also reduce the concern regarding the inefficiency of institution-based professional development programmes [62].

Based on the comprehension of the interrelatedness of the operation and development of teacher PI and metacognition, teacher educators can transform their courses into involving more teacher-as-learner-centred components, by having teachers experience the processes of problem definition, strategy design, strategy implementation, and implementation evaluation in a principled way. Teachers themselves can also explicate their daily pedagogical problem-solving processes, and try to develop their expertise through the systematic planning, performance, recording, and repetition of the problem-solving cycle in an explicit way. In addition, contextual or policy support can be added. 

Expert teachers seem to be those who constantly strive for professional development through continual and active pedagogical experimentation and meaning negotiations, with their learner and researcher identities activated. Therefore, developing these identities, which constitute PI, and activating metacognition, which seems to be the major jobs of these identities, need to be the focus of PD. The researcher hopes that future studies explore more varied cases in relation to the pedagogical problem solving of teachers of different subjects or from different cultural backgrounds, and that these studies adopt several measurements for the development of teacher metacognition and PI. This will provide further rationales to support teachers to be expert problem solvers with developed PI and metacognition.

## Figures and Tables

**Table 1:** Demographics of the participants.

Participant (Pseudonym)	Gender	Age	Teaching Experience (yrs)	Schools (in the Narratives)
Su-mi	F	Early 30s	1 ~ 5	Suburban academic high school
Eun-ju	F	Mid 30s	10 ~ 15	Suburban academic high school
Yu-na	F	Early 40s	15 ~ 20	Urban academic high school, urban vocational high school
Hei-jin	F	Late 30s	10 ~ 15	Suburban academic high school, urban academic high school
Hee-jun	M	Late 30s	10 ~ 15	Urban private academic high school

**Table 2:** Categories for data analysis.

Pedagogical Problem-Solving Procedures	PI Activation and Metacognition Operation
Problem definition	Meaning comparison for problem identification Decision whether to perform problem solving Meaning comparison and negotiation for goal setting
Strategy design	Selection of particular meanings through meaning comparison Use of analogy and creative strategy through meaning comparison and association Organisation of new strategy through meaning combination
Strategy implementation	Reflection/re-examination of the strategy and related meanings Strategy or meaning modification by regulating cognition Regulation of emotion and action to overcome frustration
Implementation evaluation	Reflection on the process and outcome of problem solving Decision to continue or discontinue problem solving Forming new meanings through repetitive experimentations

**Figure A1:**
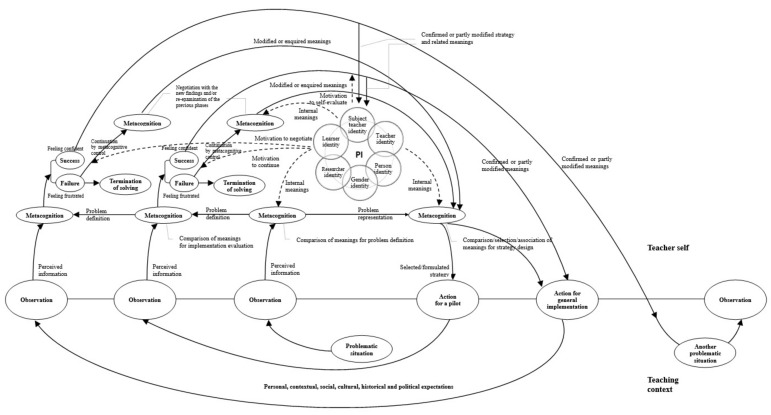
Teacher Pedagogical Problem-Solving Processes in Relation to Professional Identity and Metacognition.

## Data Availability

Not applicable.

## Appendix B

Interview Questions

1. Could you describe your teaching styles and processes?
  [relevant questions]
  - How do you plan and prepare for your lessons?
  - What materials do you use?
  - What are your roles or your students’ roles?
  - What are the challenges? And How do you handle the situations?
2. Could you describe your self-satisfactory or successful lessons?
  [relevant questions]
  - What materials or activities did you apply?
  - Why do you feel them successful?
3. Could you describe your unsatisfactory or unsuccessful lessons?
  [relevant questions]
  - Why do you feel them unsuccessful?
  - What do you think were the reasons of such outcomes?
4. What did you or do you do in order to improve your lessons?
  [relevant questions]
  - What are the challenges?
  - What do you want to learn or apply?
5. What do you think desirable pedagogies are?
  [relevant questions]
  - What do you think is important in teaching?
  - What support do you need?

## Appendix C

**Table A1:** Themes for Discussion by Participants and by Problem-Solving Phases.

	Problem Definition	Strategy Design	Strategy Implementation	Implementation Evaluation	Themes for Discussion(by Participants)
Su-mi (suburban school)	Having limited meanings for problem comprehension Problematising Ss’ cynical attitudes Valuing Ss’ concentration and comprehension	Having limited meanings about pedagogical strategies Following Eun-ju’s approach and adding her own meanings Teaching words before reading Making video material	Feeling frustrated with unexpected results Assessing the quality of her strategies through learner responses Deciding to abandon an unmanageable method Not knowing how to improve	Forming new meanings from the analysis of the findings by repeated implementations Having increased metacognition and confidence from partial success	Her insufficient internal meanings and metacognitive skills weakened problem solving. She gradually developed her metacognition and PI with problem-solving experiences (operating learner identity and interacting with Eun-ju).
Eun-ju (suburban school)	Problematising Ss’ lethargy and low academic levels Valuing Ss’ attention and comprehension Aiming to enhance Ss’ reading and vocabulary competence with several activities and materials	Using GTM familiar with her and her Ss Teaching words while reading Including group work Performing learner survey for strategy confirmation Making worksheets and PPT material	Modifying her strategy by learning from Su-mi’s findings Recognising the effectiveness of her strategy by observing Ss’ active learning and comparing the findings with her goals	Focusing on resolving the partial failure and finding out its causes through re-examination of her strategy and applied meanings Forming and reshaping her internal meanings	Her sufficient internal meanings and meaning comparison experiences benefited systematic problem solving. Her adherence to old repertoires was reduced by self-reflections, experimentations, and communications.
Yu-na (from academic school to vocational school)	Problematising test-preparation lessons in academic schools Valuing Ss’ interest and teaching language with culture Changing her context for meaning realisation	Combining different meanings for strategy design Practising theme-focused lessons based on the group work, inspired by her recent reading	Recognising the effectiveness of her strategies by observing Ss’ active learning Confirming the related meanings and forming a new meaning from the findings	Expanding internal meanings via repeated experimentations Being sceptical of applying the current meanings in academic high schools	Unavailability in meaning negotiations in academic high schools led her to avoid the endeavour for pedagogical problem solving.
Hei-jin (from suburban school to urban school)	Problematising lecture-based lessons and Ss’ low interest in learning Valuing and aiming to improve practicality, Ss’ participation, and autonomous learning	Developing text-based but task-based activities Referring to the previous teaching methods and using analogies and creativity	Repeatedly observing Ss’ active participation Confirming her strategy and applied meanings	Maintaining similar strategies and keeping experimenting	Flexible meaning negotiations in the sub-urban school led to her experiences of successful pedagogical problem solving and positive emotions.
	Problematising lecture-based lessons for test-preparation and Ss’ low concentration Aiming to control Ss’ distraction with a new strategy	Planning to give Ss a GTM-based lecture (30 min) and let them have autonomous learning with worksheets (20 min)	Modifying worksheets and management skills, based on the learner responses, with patience Observing Ss’ enhanced concentration and autonomy	Confirming her modified strategy and forming a new meaning about learning management	Confrontation of meaning gaps in urban schools led her to exercise meaning negotiations using metacognition.
Hee-jun (private school)	Valuing knowledge delivery and also learner participation and practical learning Aiming to conduct performance-oriented lessons plus several simple activities	Having and applying his established strategy of three stages: motivating for learning, lecturing, and leading Ss’ applications (old repertoire)	Assessing the effectiveness of the strategy by eye contact with Ss Partially modifying the content and material Rejecting significant changes	Being unsatisfied with his routines Performing task-based lessons but experiencing discomfort Turning back to his old repertoire for partial identity verification	He was forced to practise test preparation lessons in the private academic high school, which frustrated him and his pedagogic improvement through meaning negotiations.
Themes for discussion (by problem-solving phases)	Teachers’ sufficient internal meanings and comprehensive meaning negotiations led to specific goal setting, strategy design, and evaluations. External needs for test preparation conflict with teachers’ meanings.	Contexts play a critical role for teachers’ prioritisation of particular identities and meanings, and thus meaning negotiations for strategy design. Experienced teachers tend to use their old repertoires.	Teachers as researchers seem to precisely analyse the processes and outcomes of problem solving with metacognition, and teachers as learners seem to actively learn from the findings and modify their strategies.	Contextual requirements for test preparation can limit teachers’ further experimentations. Teachers seem to reshape their PI and its meanings and metacognition through their pedagogical problem-solving experiences.	Teacher PI and metacognition co-operate and co-develop organically through the teachers’ pedagogical problem-solving processes.
